# *Nitrosarchaeum haohaiensis* sp. Nov. CL1^T^ : Isolation and Characterisation of a Novel Ammonia‐Oxidising Archaeon From Aquatic Environments

**DOI:** 10.1111/1758-2229.70100

**Published:** 2025-05-22

**Authors:** Hailing Li, Lingqi Zhuang, Haoyun Cai, Yimin Ni, Ting Chu, Lanming Chen, Yongxin Yu, Yongjie Wang

**Affiliations:** ^1^ College of Food Science and Technology Shanghai Ocean University Shanghai China; ^2^ Laboratory for Marine Biology and Biotechnology Qingdao Marine Science and Technology Center Qingdao China

## Abstract

Following a 3.5‐year enrichment cultivation period, a novel ammonia‐oxidising archaeon (AOA), designated strain CL1^T^, was isolated from Yangshan Harbour (East China Sea). Strain CL1^T^ demonstrates a maximum ammonia tolerance of up to 10 mM. Its optimal growth conditions include a pH range of 7–8, a salinity of 2%–3%, and a temperature range of 20°C–25°C. Under these conditions, strain CL1^T^ achieved a maximum specific growth rate of 0.87 d^−1^, with cell yields estimated at 3.92 × 10^6^ cells mL^−1^ μM ammonia^−1^. Genomic sequencing revealed that strain CL1^T^ possesses a genome size of 1.63 megabases with a high completeness of 99.95%. Phylogenetic analysis based on the 16S rRNA gene and whole‐genome data placed strain CL1^T^ within the genus *Nitrosarchaeum*. The average nucleotide identity (ANI) between the genome of strain CL1^T^ and its closest relative was 92.01%, confirming that strain CL1^T^ represents a novel species within *Nitrosarchaeum*. Metabolic pathway analysis demonstrated that strain CL1^T^ encodes key enzymes for ammonia oxidation, including ammonia monooxygenase (*amoA*, *amoB*, *amoC*) and copper oxidase, indicating its capacity for ammonia oxidation. Additionally, strain CL1^T^ likely assimilates ammonia through the GS‐GOGAT and GDH pathways. Consistent with the observation of extracellular vesicles (EVs) in strain CL1^T^ via electron microscopy, genome annotation identified core genes associated with EVs function, such as *vps4* and *FtsZ*. The isolation of strain CL1^T^ provides a valuable model system for investigating its ammonia metabolism and exploring its ecological interactions with other AOA, ammonia‐oxidising bacteria (AOB) and nitrite‐oxidising bacteria (NOB), thereby contributing to a deeper understanding of nitrogen cycling mechanisms in aquatic environments.

## Graphical abstract

Following a 3.5‐year enrichment cultivation period, a rapid‐growing ammonia‐oxidising archaeon, designated CL1^T^, was isolated from the East China Sea. Strain CL1^T^ represents a new species within the *Nitrosarchaeum* genus and is capable of effectively oxidising ammonia at both low and high concentrations through the GS‐GOGAT and GDH pathways. Furthermore, the genome of strain CL1^T^ encodes core genes associated with extracellular vesicles (EVs), including *vps4* and *FtsZ*, as evidenced by the observation of EVs of strain CL1^T^ using electron microscopes.

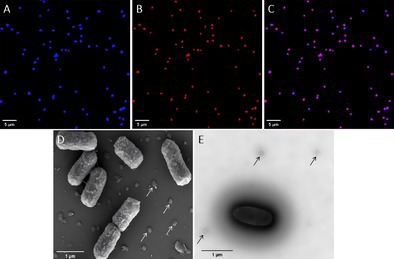

## 1 Introduction

In the mesopelagic and bathypelagic zones of the ocean, archaea constitute a significant proportion of marine planktonic microbial communities, representing 20%–40% of the total population (Karner et al. 2001; Teira et al. 2004, 2006). Marine planktonic archaea are broadly categorised into four major phylogenetic groups: Marine Group I (MGI, classified within the phylum *Nitrososphaerota*, formerly known as *Thaumarchaeota*) (DeLong 1992), Marine Group II (MGII) (DeLong 1992), Marine Group III (MGIII) (Fuhrman and Davis 1997), and Marine Group IV (MGIV) (López‐García et al. 2001). Each of these groups exhibits unique physiological and ecological traits, reflecting their specialised roles in marine ecosystems (Santoro et al. 2019). Amongst these, the first cultured representative of MGI, *Nitrosopumilus maritimus* SCM1^T^, is a chemolithoautotrophic organism that derives energy through ammonia oxidation, thereby classified as an ammonia‐oxidising archaeon (AOA) (Könneke et al. 2005). AOA are globally distributed across diverse ecosystems, showcasing remarkable adaptability to varying environmental conditions and nutrient availability (Francis et al. 2005).

In 2012, a landmark analysis of the *amoA* gene enabled the classification of AOA into five major clusters (Pester et al. 2012): the *Nitrosopumilus* cluster (previously termed the marine or I.1a AOA lineage (DeLong 1998)), the *Nitrososphaera* cluster (formerly known as the soil or I.1b AOA lineage (DeLong 1998)), *Nitrosocaldus* (initially identified as ThAOA or HWCGIII lineage (de la Torre et al. 2008; Prosser and Nicol 2008)), *Nitrosotalea* (also linked to group I.1a AOA (Lehtovirta‐Morley et al. 2011)), and the *Nitrososphaera* sister cluster (a previously unrecognised group). The most recent taxonomic framework for AOA organises them into four primary orders: *Nitrosopumilales*, *Nitrososphaerales*, *Candidatus* (*Ca*.) Nitrosomirales, and *Ca*. Nitrosocaldales (Zheng et al. 2024). Members of the *Nitrosopumilales* are predominantly found in aquatic environments, including both marine and freshwater ecosystems (Zheng et al. 2024) (Nunoura et al. 2015). In contrast, *Nitrososphaerales* are primarily associated with terrestrial habitats such as grassland and hillside soils, as well as aquifer sediments (Diamond et al. 2022). *Ca*. Nitrosocaldales exhibit specialised adaptations to thrive in geothermal environments, reflecting their unique evolutionary trajectory (Luo et al. 2024). The recently described order *Ca*. Nitrosomirales demonstrates remarkable ecological versatility, with members distributed across diverse habitats, including groundwater, geothermal sites, and both terrestrial and marine environments (Zheng et al. 2024). This taxonomic and ecological diversity underscores the extraordinary adaptability of AOA, enabling them to occupy and drive nitrogen cycling in a wide array of ecosystems worldwide.

*Nitrososphaera viennensis* EN76^T^ was isolated from soil, representing the first cultured strain within the *Nitrososphaera* genus (Tourna et al. 2011). Unlike SCM1^T^, EN76^T^ can utilise both ammonia and urea as nitrogen sources for growth, although it requires higher ammonia concentrations to sustain its metabolic activity (Tourna et al. 2011). Further expanding the diversity of cultured AOA, *Nitrosarchaeum koreense* MY1^T^ was isolated from agricultural soil, marking the first representative of the *Nitrosarchaeum* genus (Jung et al. 2018). Most recently, an ammonia‐oxidising enrichment culture (BO1) was obtained from the sediment of Lake Burr Oak, a freshwater reservoir in Ohio, USA, and high‐quality metagenome‐assembled genomes (MAGs) were reconstructed from BO1, revealing affiliations with the genera *Nitrosarchaeum* and *Nitrospira* (Bollmann et al. 2024).

Yangshan Harbour, located approximately 30 km southeast of Shanghai in the East China Sea, is a region of significant ecological complexity due to its unique hydrological environment. This area is influenced by the convergence of the Yangtze River, Qiantang River and the East China Sea, resulting in water characterised by high sediment loads, dynamic hydrodynamic conditions and variable water quality. In our previous study, we demonstrated that *Nitrososphaerota* dominates the archaeal community in Yangshan Harbour, accounting for 84.1% of the total archaeal population (Zhou et al. 2023). Additionally, we identified and characterised genomic sequences of 26 novel AOA viruses from the same water samples, shedding light on the viral diversity and its potential interactions with AOA in this ecosystem (Zhou et al. 2023; Ni et al. 2024). These findings underscore the ecological significance of Yangshan Harbour as a hotspot for studying archaeal diversity, viral ecology, and their roles in nitrogen cycling within complex aquatic environments.

In this study, water samples were collected from Yangshan Harbour with the aim of enriching and isolating AOA. Following an extensive 3.5‐year enrichment cultivation process, a novel ammonia‐oxidising archaeon, designated as strain CL1^T^, was successfully isolated. This achievement represents the first pure culture of a *Nitrosarchaeum* species derived from an aquatic environment. Genomic analysis of strain CL1^T^ reveals that it possesses core genes involved in ammonia oxidation, including *amoA*, *amoB* and *amoC*, which encode the key subunits of ammonia monooxygenase. Additionally, strain CL1^T^ harbours essential genes associated with a dual ammonia assimilation pathway, enabling it to incorporate nitrogen into biomass through multiple metabolic routes. These genetic features highlight strain CL1^T^'s metabolic versatility and its ability to thrive in diverse environmental conditions. The discovery of strain CL1^T^ provides a foundation for further investigation into the physiological characteristics of AOA and their role in aquatic nitrogen cycling.

## 2 Materials and Methods

### 2.1 Sample Source and Culture Conditions

Water samples were collected from Yangshan Harbour in the East China Sea (Shanghai, China) (Table 1), with three sampling locations selected: Da Yangshan, Xiao Yangshan and the Central Channel. A 5‐L water sampler was employed to collect water samples from three distinct depths of 1, 5 and 8 m, with the objective of minimising potential biases from a single depth. A total of 45 L of water was collected, with 15 L obtained from each location.

**TABLE 1:** Water samples obtained from Yangshan Harbour.

Sampling sites	Longitude and latitude	Temperature (°C)	pH	Salinity (‰)	Conductivity (mS)	Total dissolved solids (g/L)	Sampling depth (m)
Da Yangshan	30°37′0″ N	23	7.7	23.0	30.5	26.5	1, 3, 8
	122°6′0″ E						
Central channel	30°36′17″ N	23.5	7.4	23.5	31.2	22.1	
	122°5′25″ E						
Xiao Yangshan	30°36′35″ N	23.6	7.3	22.7	32.0	21.4	
	122°7′5″ E						

The samples were promptly transferred to the laboratory at 4°C. Thereafter, they were left to stand for 4 h to allow the sediment to settle. The larger sediment particles were removed by filtering through cheesecloth, and the remaining water was filtered using a vacuum filtration system. The removal of plankton was facilitated by a 5 μm pore size cellulose nitrate membrane, followed by filtration through a 0.22 μm pore size membrane to capture prokaryotes. The filter with retained microorganisms was then cut into pieces and added to 50 mL of AOA liquid culture medium (see below) for enrichment cultivation. To ensure a sufficient sample size for subsequent analysis, 20 batches were prepared.

The formulation of the AOA liquid culture medium was based on the water quality parameters of Yangshan Harbour (Table 1) and references (Könneke et al. 2005; Tourna et al. 2011; Kim et al. 2016; Li et al. 2016; Sauder et al. 2017, 2018; Jung et al. 2018; Zou et al. 2023). A basic salt solution was prepared with the following concentrations: NaCl (20 g L^−1^), MgSO_4_ (2.44 g L^−1^), MgCl_2_·6H_2_O (5 g L^−1^), CaCl_2_ (1.1 g L^−1^), and KBr (0.1 g L^−1^). The solution was then sterilised by heating at 121°C for 20 min. Following this, heat‐sensitive reagents were added, including CH_3_COCOONa (0.1 mM), NH_4_Cl (1 mM), NaHCO_3_ (2 mM), KH_2_PO_4_ (2 mg L^−1^), and FeNaEDTA (7.5 μM). A trace element solution was subsequently prepared, containing HCl (100 mM), H_3_BO_3_ (30 mg L^−1^), CoCl_2_·6H_2_O (90 mg L^−1^), CuCl_2_·2H_2_O (2 mg L^−1^), NiCl_2_·6H_2_O (24 mg L^−1^), Na_2_MoO_4_·2H_2_O (36 mg L^−1^), ZnSO_4_·7H_2_O (144 mg L^−1^) and MnCl_2_·4H_2_O (100 mg L^−1^). After thoroughly mixing the trace element solution, 1 mL was added to 50 mL of the culture medium. In order to inhibit bacterial growth, the medium was supplemented with 50 μM ampicillin or kanamycin, alternating between the two antibiotics (Park et al. 2010; Jung et al. 2018; Tu et al. 2019). The pH was adjusted to between 7.0 and 8.0 using NaOH and HCl (1 M). The culture medium was then sterilised through a 0.22 μm pore size syringe filter.

AOA were cultured in the dark at 25°C, with the headspace of the culture bottles maintained at 30%–60% to ensure optimal conditions for AOA growth (Imachi et al. 2011; Jung et al. 2011; Lehtovirta‐Morley et al. 2011). Once the AOA reached the stationary phase, the culture was filtered through a 0.45 μm pore size filter to remove bacteria and was then transferred to a fresh medium at a 5% (v/v) inoculation rate (Park et al. 2010; Santoro and Casciotti 2011; Bayer et al. 2016).

During the isolation process, a combination of continuous dilution and end‐point dilution techniques was employed to effectively separate and purify the target archaea. To selectively suppress the growth of ammonia‐oxidising bacteria and nitrite‐oxidising bacteria, the medium was supplemented with 50 μM allylthiourea and 50 μM chlorate, respectively, as previously described (Jung et al. 2018). Contamination tests were conducted by PCR, qPCR, fluorescence in situ hybridization, as well as by inoculating the culture into diluted LB medium for heterotrophic microorganisms.

### 2.2 Growth Monitoring

The concentration of ammonium ions in the system was quantified using the Ammonium Ion Detection Kit (TIANGEN, Shanghai) following the indophenol blue colorimetric method. This approach relies on the reaction of ammonium ions with phenol and sodium hypochlorite in a highly alkaline environment, producing the water‐soluble dye indophenol blue. This compound exhibits a characteristic absorption peak at 625 nm, with the absorbance intensity being directly proportional to the ammonium ion concentration. In the experimental setup, test tubes, blank tubes, and standard tubes were prepared. After the addition of detection reagents and thorough mixing, the samples were incubated at room temperature for 1 h. The absorbance at 625 nm was then measured using a spectrophotometer to determine the ammonium ion concentration.

$${\mathrm{NH}}_{4}^{+}\text{content}\;\left(\mathrm{mM}\right)=\frac{0.22\times \left(△A+0.0063\right)\times D}{V\times 18.04}$$

where Δ*A* = *A*_test_ − *A*_blank_, *D* represents the dilution factor, *V* is the sample volume added (mL), 18.04 is the molecular weight of the ammonium ion, and the values 0.22 and 0.0063 are calculated from the standard curve with *R* ^2^ = 0.9998.

The nitrite content in the culture medium was determined in accordance with the instructions of the Nitrite Detection Kit (Sangon, Shanghai). At two‐day intervals, 25 μL of the culture sample was collected and placed into a test tube, with blank and standard tubes established as controls. After adding the detection reagent, the mixture was thoroughly mixed and allowed to stand for 15 min. Subsequently, 200 μL of the mixed solution was transferred to a 96‐well plate for the measurement of its optical density at 540 nm (A540). The nitrite concentration in the sample was calculated using the following formula, which was used to assess the growth and metabolic activity of AOA.

$$\text{Nitrite content}\;\left(\mathrm{mM}\right)=\frac{0.04\times \left(A_{\text{sample}}-A_{\text{blank}}\right)}{A_{\text{Standard}}-A_{\text{blank}}}$$

where 0.04 indicates the concentration of the standard solution at 0.04 μmol/mL.

To evaluate the ammonium tolerance of strain CL1^T^, ammonium chloride was added to the culture medium at concentrations of 0, 0.5, 1, 3, 5, 10 and 20 mM. The pH of the medium was adjusted to 5, 6, 7, 8, 9 and 10 using 1 M HCl and 5 M NaOH to determine the optimal pH for strain CL1^T^ growth. The effect of NaCl concentration on strain CL1^T^ growth was analysed by supplementing the medium with 0%, 0.5%, 1%, 2%, 3%, 5% and 10% (w/v) NaCl. To identify the optimal growth temperature, strain CL1^T^ was incubated at 15°C, 20°C, 25°C, 30°C, 35°C and 40°C. In all experiments, strain CL1^T^ was inoculated at 5% (v/v) (50 mL medium), and all other medium components and culture conditions were consistent with those used in the enrichment culture. The cultures were maintained for 21 days, with sampling and analysis performed every 3 days. The ammonium tolerance of strain CL1^T^ and the effects of pH, salinity and temperature on its ammonia metabolism were assessed by measuring nitrite concentration.

All experimental conditions were tested in three biological replicates to account for variability and ensure robust results.

### 2.3 DNA Extraction

At two‐day intervals, 1 mL of the culture sample was filtered using a sterilised Millipore mixed cellulose ester filter membrane (0.22 μm pore size) to collect the archaeal biomass. Subsequently, the filter membrane was minced into small pieces, and the DNA was extracted according to the protocol outlined in the FastDNA Spin Kit for Soil (MP Biomedicals, USA). The concentration and integrity of the extracted genomic DNA were then assessed using a multifunctional microplate reader (BioTek Synergy 2) and 1% agarose gel electrophoresis.

### 2.4 PCR And Sequencing

The enrichment of AOA in the culture was analysed by PCR detection of the archaeal 16S rRNA gene, archaeal *amoA* gene and bacterial 16S rRNA gene (Table 2). The PCR reaction mixture comprised 25 μL, including 12.5 μL of 2× *Taq* Master Mix buffer (Vazyme, Nanjing), 1 μL of each 10 μM forward and reverse primers, 1 μL of DNA template, and ddH_2_O to a final volume of 25 μL. The PCR thermal cycling conditions were as follows: an initial denaturation at 95°C for 3 min, followed by 30 cycles of 95°C for 15 s, 60°C for 15 s, and 72°C for 15 s, with a final extension at 72°C for 5 min. PCR products were analysed by 1% agarose gel electrophoresis (120 V, 400 mA) to assess the amplification of the target gene.

**TABLE 2:** Primers used in PCR and qPCR.

Target Gene	Amplification length (bp)	Primer	Sequence (5′ → 3′)	References
Archaea *amoA*	635	AmoAF	STAATGGTCTGGCTTAGACG	(Francis et al. 2005)
		AmoAR	GCGGCCATCCATCTGTATGT	
Archaea 16S rRNA	938	Arch20F	TTCCGGTTGATCCYGCCRG	(Park et al. 2010)
		Arch958R	TCCGGCGTTGAMTCCAATT	
Bacteria 16S rRNA	1465	27F	AGAGTTTGATCMTGGCTCAG	
		1492R	GGYTACCTTGTTACGACTT	
Archaea 16S rRNA	208	519F	CAGCMGCCGCGGTAA	
		727R	GCTTTCRTCCCTCACCGT	

The PCR products of the archaeal 16S rRNA and archaeal *amoA* genes were subjected to Sanger sequencing. The sequences were then analysed using the NCBI online BLASTN tool (https://blast.ncbi.nlm.nih.gov/) against the nucleotide collection (nr/nt) database, with the database last updated on November 20th, 2024. The following parameters were applied: max target sequences = 100, expect threshold = 0.05, word size = 28, and match/mismatch scores = 1 and −2.

### 2.5 qPCR Analysis

The fluorescence‐based quantitative PCR (qPCR) was performed for absolute quantification of archaea in the enriched cultures. First, a plasmid standard containing a fragment of the 16S rRNA gene was constructed. The archaeal 16S rRNA gene fragment was purified from the PCR products and ligated into the pUCM‐T plasmid vector (pUCM‐T ligation kit, Sangon, Shanghai). The recombinant plasmid was then introduced into *Escherichia coli* DH5α competent cells via heat shock. Positive clones were identified by blue‐white colony screening and PCR verification, and the positive colonies were cultured in an LB liquid medium containing ampicillin (37°C, 200 rpm, 12 h). The extracted and purified plasmid DNA was subsequently quantified using a dsDNA HS Assay Kit Qubit (YESEN, Shanghai) to a final concentration of 7.5 × 10^9^ copies/μL.

The qPCR reaction mixture comprised 20 μL of solution, including 10 μL of 2× ChamQ Universal SYBR qPCR Master Mix (Vazyme, Nanjing), 0.4 μL of 10 μM forward and reverse primers (Table 2), 1 μL of template DNA, and ddH_2_O to a final volume of 20 μL. The reaction program included an initial denaturation step at 95°C for 30 s, followed by 40 amplification cycles consisting of 95°C for 10 s and 60°C for 30 s. The melt curve analysis was performed with the following steps: 95°C for 15 s, 60°C for 60 s and 95°C for 15 s. The slope of the standard curve, which was generated using the archaeal 16S rRNA gene standard curve equation (*y* = −3.15968*x* + 36.9450), is approximately −3.16, with an efficiency of 107% and an *R* ^2^ value of 0.993. These values satisfy the criteria for a valid standard curve. Triplicate wells were prepared for each sample.

### 2.6 Fluorescence In Situ Hybridization

200 μL of culture in the stationary phase was collected, diluted with sterile water to a final volume of 1 mL, and then mixed with 1 mL of Gluta fixative (2.5%). The mixture was fixed at room temperature for 1 h. Following this, the sample was filtered through a 0.2 μm pore size, 25 mm diameter Al_2_O_3_ membrane (Whatman, United Kingdom). Hybridisation was performed with a Cy3‐labelled archaeal‐specific probe (Arch915) (Alm et al. 1996) and a FAM‐labelled bacterial‐specific probe (EUB338) (Amann et al. 1990). To visualise all cells, a counterstaining step was carried out using 4′,6‐diamidino‐2‐phenylindole (DAPI). Finally, the samples were observed under a 63× oil immersion lens using a Leica TCS SP8 laser confocal microscope.

### 2.7 Scanning Electron Microscopy and Transmission Electron Microscopy

40 mL of enriched culture at the stationary phase was collected and centrifuged to concentrate the sample to 1 mL. 1 mL of Gluta fixative (2.5%) was then added, and the sample was stored overnight at 4°C. After fixation, the samples were washed three times with 0.1 M phosphate‐buffered saline (PBS, pH 7.4), with each wash lasting 15 min. Subsequently, under dark conditions, the samples were fixed with 1% osmium tetroxide (prepared in 0.1 M PBS) at room temperature for 1–2 h, followed by three additional washes with PBS. The fixed cells were dehydrated in a graded ethanol series: 30%, 50%, 70%, 80%, 90% and 95% ethanol for 15 min each. The samples were then infiltrated with isoamyl acetate for 15 min to replace the ethanol. After dehydration, the samples were air‐dried and mounted onto conductive carbon tape. Gold sputtering was performed for approximately 30 s using an ion sputter coater. Finally, the samples were observed using a scanning electron microscope (SEM, HITACHI Regulus 8100).

1 mL of enriched culture in the stationary phase was collected and centrifuged to concentrate the sample to 200 μL. Subsequently, 200 μL of Gluta fixative (2.5%) was added, and the sample was stored overnight at 4°C. A 20 μL aliquot of the cell suspension was placed on a carbon‐coated copper grid and allowed to sit for 3–5 min. The excess liquid was then blotted off with filter paper. The cells were then stained with 2% phosphotungstic acid for 1–2 min. After air‐drying at room temperature, the cells were observed using a transmission electron microscope (TEM, HITACHI HT7700), and images were captured for further analysis.

### 2.8 Genomic Sequencing and Analysis

DNA was extracted (see Section 2.3) and amplified using the MDA Fluorescent Dye‐based Real‐Time Whole Genome Amplification Kit (XinYu Biotechnology, Shanghai). The amplified DNA was then used for library construction and sequencing at Guangdong MegGene Technology Co. Ltd.

Quality control was performed on the reads using Trimmomatic software (Bolger et al. 2014) with the following parameters: Leading: 3, Trailing: 3, SlidingWindow: 5:20, Minlen: 50. The clean reads were then assembled de novo using Megahit (Li et al. 2015), with K‐mer parameters set to *k*‐min 35, *k*‐max 95, and *k*‐step 20. The resulting scaffolds were fragmented at one or more consecutive N positions to generate scaffold fragments (scaftigs). Scaftigs shorter than 500 bp were filtered out. Genome binning was performed using MetaBin G2 (Choi et al. 2021), based on GC content and K‐mer frequency. The binned genomes were classified and annotated using GTDB‐Tk (Chaumeil et al. 2019). Finally, genome completeness and contamination were assessed using CheckM (Parks et al. 2015), which evaluates the quality of the binned genomes by calculating their completeness and contamination. Prokka (v1.14.6) (Seemann 2014) was used to predict coding sequences and assign functional annotations, with reference to UniProt and COG databases. The analysis was performed with default parameters, referencing its built‐in databases, including UniProt (default version at installation), rRNA/tRNA, Pfam and COG databases.

The genome sequence of strain CL1^T^ was analysed using the Type (Strain) Genome Server (TYGS) (https://tygs.dsmz.de) (Meier‐Kolthoff and Goker 2019; Meier‐Kolthoff et al. 2022). Closely related type strains were identified based on MASH genome comparisons (Ondov et al. 2016) and BLAST analysis of 16S rRNA sequences extracted with RNAmmer (Lagesen et al. 2007). Pairwise intergenomic distances were calculated using the Genome BLAST Distance Phylogeny (GBDP) approach with the distance formula d5 (Meier‐Kolthoff et al. 2014), and digital DNA–DNA hybridisation (dDDH) values were used for species and subspecies clustering (thresholds: 70% and 79%) (Meier‐Kolthoff et al. 2014). Phylogenetic trees were inferred with FastME 2.1.6.1 (Lefort et al. 2015) based on GBDP distances, using 100 pseudo‐bootstrap replicates for branch support. The 16S rRNA tree and genome‐based tree were rooted at the midpoint (Farris 1972) and visualised with Chiplot (https://www.chiplot.online/) (Xie et al. 2023).

Genomes from the *Nitrosarchaeum* genus with completeness > 85%, as well as genomes from the *Nitrosopumilus* genus with available pure culture strains, were first selected based on GTDB classification. For Average Nucleotide Identity (ANI) analysis, FastANI (Hernandez‐Salmeron and Moreno‐Hagelsieb 2022) (https://github.com/ParBLiSS/FastANI, v1.34) was used to calculate the ANI values of the target genomes, generating an ANI matrix file. The resulting matrix was then imported into TBtools (Chen et al. 2023) (https://github.com/CJ‐Chen/TBtools‐Manual, v2.121), where the built‐in matrix visualisation function was used to create graphical representations of the results.

The KEGG Automatic Annotation Server (KAAS) (https://www.genome.jp/kegg/kaas/) (Moriya et al. 2007) was used to annotate the genome and predict metabolic pathways. The presence of CRISPR‐Cas systems in the genome of strain CL1^T^ was predicted using CRISPRCasFinder (v4.2.20) (Couvin et al. 2018). The analysis was conducted with default parameters, including spacer length ≥ 18 bp and repeat length ≥ 21 bp, and utilised its built‐in database for Cas protein gene identification.

To determine the phylogenetic placement of strain CL1^T^'s Cas1 protein, Cas1 sequences from the study referenced in (Krupovic et al. 2014) were downloaded and combined with the Cas1 sequence of strain CL1^T^. Additionally, using strain CL1^T^'s Cas1 as a query, BLASTp searches were performed against the NCBI archaeal nr database. The resulting sequences were processed with CD‐HIT (Li and Godzik 2006) to remove redundancy, with sequences retained if they had a similarity > 65%. All sequences were then aligned using MAFFT (v7.526) (Rozewicki et al. 2019), and a phylogenetic tree was constructed with FastTree 2.1 (Price et al. 2010) based on the aligned sequences. The resulting tree was visualised using the iTOL (Interactive Tree of Life) tool (Letunic and Bork 2007).

## 3 Results and Discussion

### 3.1 Enrichment of AOA in Yangshan Harbour Water

AOA are responsible for the conversion of NH_4_^+^ to NO_2_^−^ during the ammonia oxidation process (Kraft et al. 2022). The production of NO_2_^−^ is closely linked to the cell growth cycle (Qin et al. 2014; Chen et al. 2017; Jung et al. 2018). The concentration of NO_2_^−^ reaching 0.8–1.0 mM indicates that the nitrogen source in the medium is nearly exhausted (Park et al. 2010; Jung et al. 2014), thereby initiating the stationary phase of microbial growth and necessitating subculturing.

**FIGURE 1:**
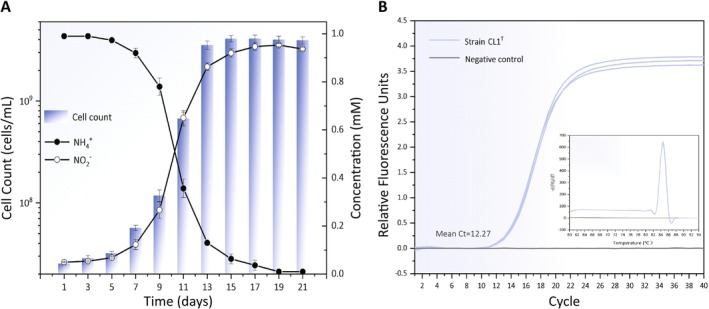
The growth curve of the culture strain CL1^T^. (A) The line chart depicts the concentrations of ammonium ions and nitrite, respectively. The bar chart illustrates the cell count, as determined by qPCR targeting the archaeal 16S rRNA gene. Error bars on both charts represent the standard deviations derived from triplicate experiments. (B) Abundance of strain CL1^T^ by the 13th day of growth. The melting curves are displayed alongside the amplification curves, with the ordinate, d(Ft)/dT, representing the negative first derivative of the fluorescence signal intensity with respect to temperature. The blue line corresponds to strain CL1^T^, whilst the grey line represents the negative control. Three parallel wells were used in the analysis.

In this study, the growth dynamics of AOA in the enriched culture were monitored by measuring nitrite concentrations and quantifying the abundance of archaeal 16S rRNA genes at two‐day intervals. After 3.5 years of serial subculturing, followed by a combination of continuous dilution and end‐point dilution, a culture (designated strain CL1^T^; unless otherwise specified, all subsequent analyses were performed using the strain CL1^T^ culture) with robust ammonia oxidation capacity was successfully obtained. Between days 5 and 13 of cultivation, both nitrite concentration and archaeal 16S rRNA gene abundance showed a rapid increase, after which growth decelerated and eventually stabilised (Figure 1A). The depletion of ammonia and the accumulation of nitrite exhibited an inverse correlation (Figure 1A), indicating that strain CL1^T^ oxidises ammonia to generate energy for cell growth, with nitrite serving as the primary metabolic end product. By the 13th day, the nitrite concentration reached 0.8–0.9 mM, demonstrating that the initial 1 mM ammonium chloride energy source was nearly exhausted and that strain CL1^T^ had entered the stationary growth phase. At this point, the archaeal 16S rRNA gene copy number of strain CL1^T^ was determined to be 3.55 × 10^9^ ± 3.36 × 10^8^ copies/mL (Figure 1). Additionally, the melt curve analysis of strain CL1^T^ revealed a single sharp peak (Figure 1B), confirming the homogeneity of the amplification products. This finding strongly suggests that strain CL1^T^ represents a single species of AOA, highlighting the success of the enrichment and isolation process.

### 3.2 Purity of the Strain CL1^T^

**FIGURE 2:**
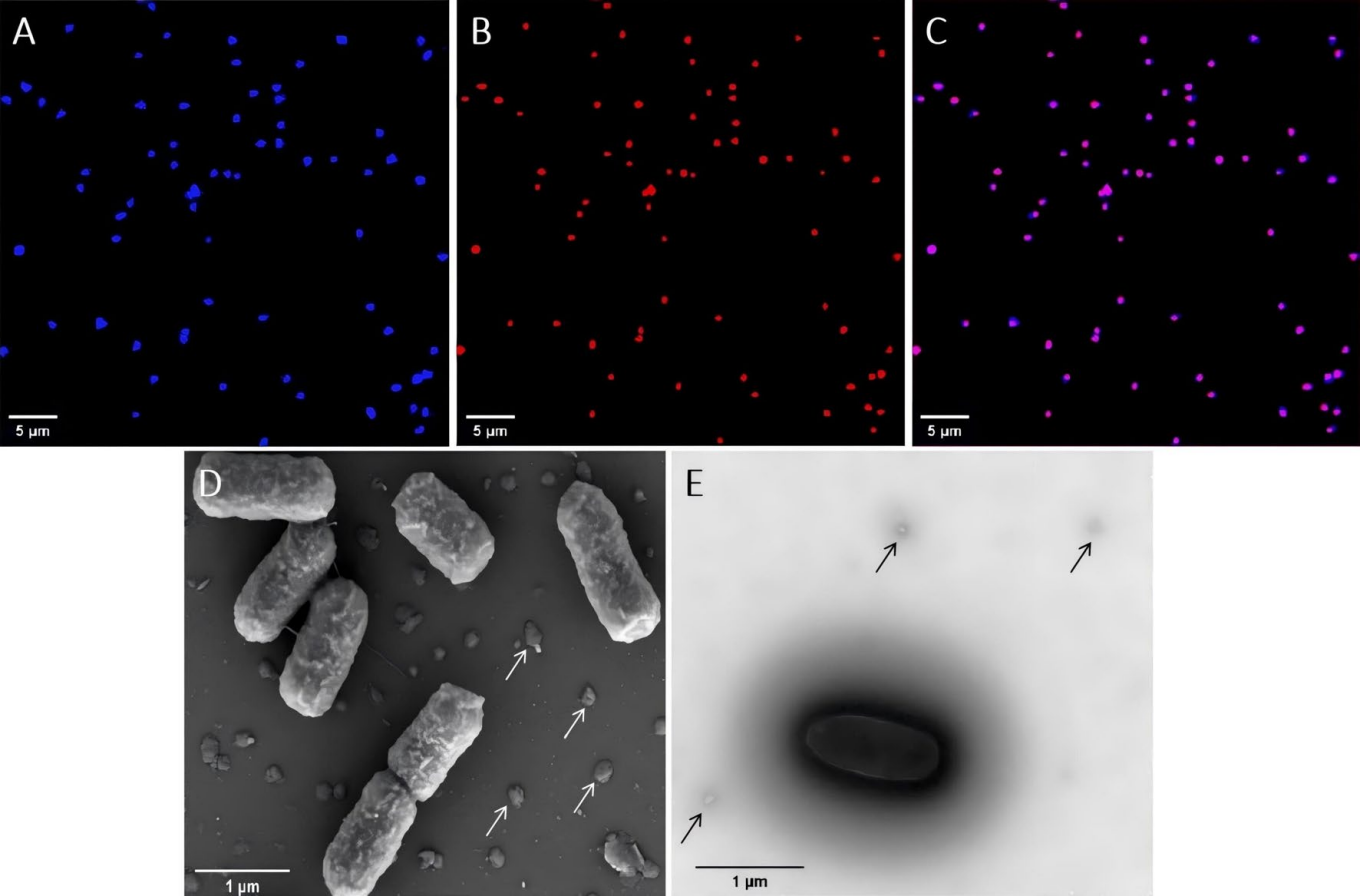
Microscopic examination of strain CL1^T^ cells. (A) Epifluorescence microscopic images of cells stained with DAPI (blue). (B) FISH image of Cy3‐labelled Archaea‐specific probe (Arc915, red). (C) Merged image of A and B. (D) Scanning electron and (E) Transmission electron micrographs of strain CL1^T^ cells. The arrow indicates the potential EVs.

To confirm the purity of the strain CL1^T^ culture, we performed fluorescence in situ hybridisation (FISH) analysis on a stationary‐phase strain CL1^T^ sample (cultivated for 13 days). The results revealed that all cells stained with DAPI (Figure 2A) successfully hybridised with an archaeal‐specific probe (Figure 2B,C), whilst no hybridisation was observed with a bacterial‐specific probe (data not shown). These findings conclusively demonstrate that strain CL1^T^ is exclusively composed of archaeal cells, with no detectable bacterial contamination.

**FIGURE 3:**
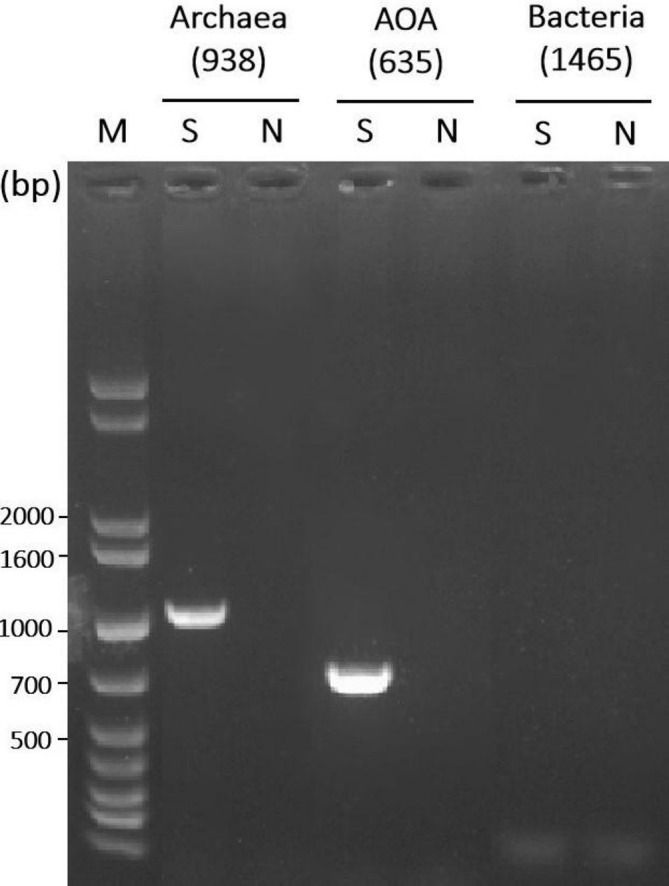
Agarose gel electrophoresis of PCR amplicon derived from strain CL1^T^. M, S and N denote the 1 kb Plus DNA Marker, the DNA from the strain CL1^T^ culture, and the negative control, respectively. The PCR products for the archaeal 16S rRNA gene, AOA ammonia monooxygenase gene (*amoA*), and bacterial 16S rRNA gene were separated on 1% agarose gel. The expected target sizes for these products are 938, 635 and 1465 bp, respectively.

To further validate the purity and taxonomic identity of the strain CL1^T^ culture, DNA was extracted from a stationary‐phase strain CL1^T^ sample (cultivated for 13 days) and subjected to PCR amplification and sequencing analysis. The PCR products of the archaeal 16S rRNA and archaeal *amoA* genes displayed distinct, single bands on the agarose gel, with sizes corresponding to the expected target product lengths (Figure 3). Consistent with the FISH findings, no amplification products of the bacterial 16S rRNA gene were detected (Figure 3). The PCR amplification products of the archaeal 16S rRNA and *amoA* genes were sequenced, yielding single sequences of 852 bp (PQ498924) and 574 bp (SRR31438651), respectively. Sequence alignment with the NCBI database revealed that the 16S rRNA gene sequence of strain CL1^T^ exhibited 99% coverage and 99.65% similarity to that of *Nitrosarchaeum koreense* MY1^T^ (NR 177299.1). Similarly, the *amoA* gene sequence showed 100% coverage and 98.95% similarity to the same reference strain. These findings conclusively demonstrate that strain CL1^T^ represents a single AOA species within the *Nitrosarchaeum* genus.

Additionally, the strain CL1^T^ culture was inoculated into diluted LB medium. No growth was observed under these conditions, confirming the absence of contaminating heterotrophic microorganisms in the strain CL1^T^ culture.

### 3.3 Morphology and Physiological Characteristics of Strain CL1^T^

SEM (Figure 2D) and TEM (Figure 2E) analyses revealed that strain CL1^T^ is a short rod‐shaped archaeon, measuring 1.2–1.4 μm in length and 0.5–0.7 μm in width. Notably, no pili or archaellum were observed on its surface. In comparison, the MY1^T^ exhibits dimensions of 0.6–1.0 μm in length and 0.3–0.6 μm in width, the SCM1^T^ measures 0.5–0.9 μm in length and 0.17–0.22 μm in width, and *Nitrosotenuis aquarius* AQ6F^T^ ranges from 0.6–3.6 μm in length and 0.4 μm in width. All three strains share a rod‐shaped morphology, and similar to strain CL1^T^, no pili or archaellum have been observed under electron microscopy (Könneke et al. 2005; Jung et al. 2018; Sauder et al. 2018) (Table 3). These observations indicate that strain CL1^T^'s morphology, size, and lack of extracellular appendages are consistent with those of closely related AOA species.

**TABLE 3:** Major physiological features of strain CL1^T^ compared with those of other related representatives.

Characteristic	CL1^T^	MY1^T^ (Jung et al. 2018)	SCM1^T^ (Könneke et al. 2005)	AQ6F^T^ (Sauder et al. 2018)	HCA1^T^ (Qin et al. 2014, 2017)	HCE1^T^ (Qin et al. 2017)	PS0^T^ (Qin et al. 2014, 2017)
Ammonium tolerance (mM)	10	10	10	3	10	1	20
Growth pH:							
Range	6.0–8.0	6.0–8.0	6.8–8.1	ND	6.8–8.1	6.4–7.8	5.9–8.1
Optimum	7.0–8.0	7.0	7.3		7.3	7.3	6.8
Growth salinity (%):							
Range	0.5–5.0	0.1–0.6	1.6–5.5	0.001–0.05	1.5–4.0	1.0–4.0	1.5–4.0
Optimum	2.0–3.0	0.2–0.4	3.2–3.7	0.05	3.2	2.5–3.2	2.5
Growth temperature (°C):							
Range	20–30	15–30	15–35	20–40	10–30	4–30	10–30
Optimum	25–30	25	32	30–33	25	25	26
The cell yield (cells mL^−1^ μM ammonia^−1^)	3.92 × 10^6^	2.4 × 10^5^	2.7 × 10^4^	ND	ND	ND	ND
Maximum specific growth rate (day^−1^)	0.87	0.43	0.78	ND	0.55	0.43	0.23
DNA G + C content (mol%)	32.84	32.7	34.2	42.2	33.0	33.1	33.4
Cell shape, diameter and length (μm)	Short rod	Short rod	Short rod	Rod	Rod	Rod	Rod
	0.5–0.7	0.3–0.6	0.17–0.22	0.4	0.15–0.26	0.15–0.26	0.15–0.26
	1.2–1.4	0.6–1.0	0.5–0.9	0.6–3.6	0.50–1.59	0.50–1.59	0.50–1.59

Abbreviation: ND, no data.

Interestingly, the electron micrographs of strain CL1^T^ (Figure 2D,E) suggest the presence of extracellular vesicles (EVs), resembling those previously documented in extremophilic members of the *Euryarchaeota* and *Crenarchaeota* phyla (Liu et al. 2021). To date, no other AOA has been reported to produce EVs, making strain CL1^T^ a unique candidate for studying EVs biology in non‐extremophilic archaea. The discovery of EVs in strain CL1^T^ opens new avenues for investigating their potential roles in environmental interactions, stress protection, genetic material exchange and metabolic processes.

**FIGURE 4:**
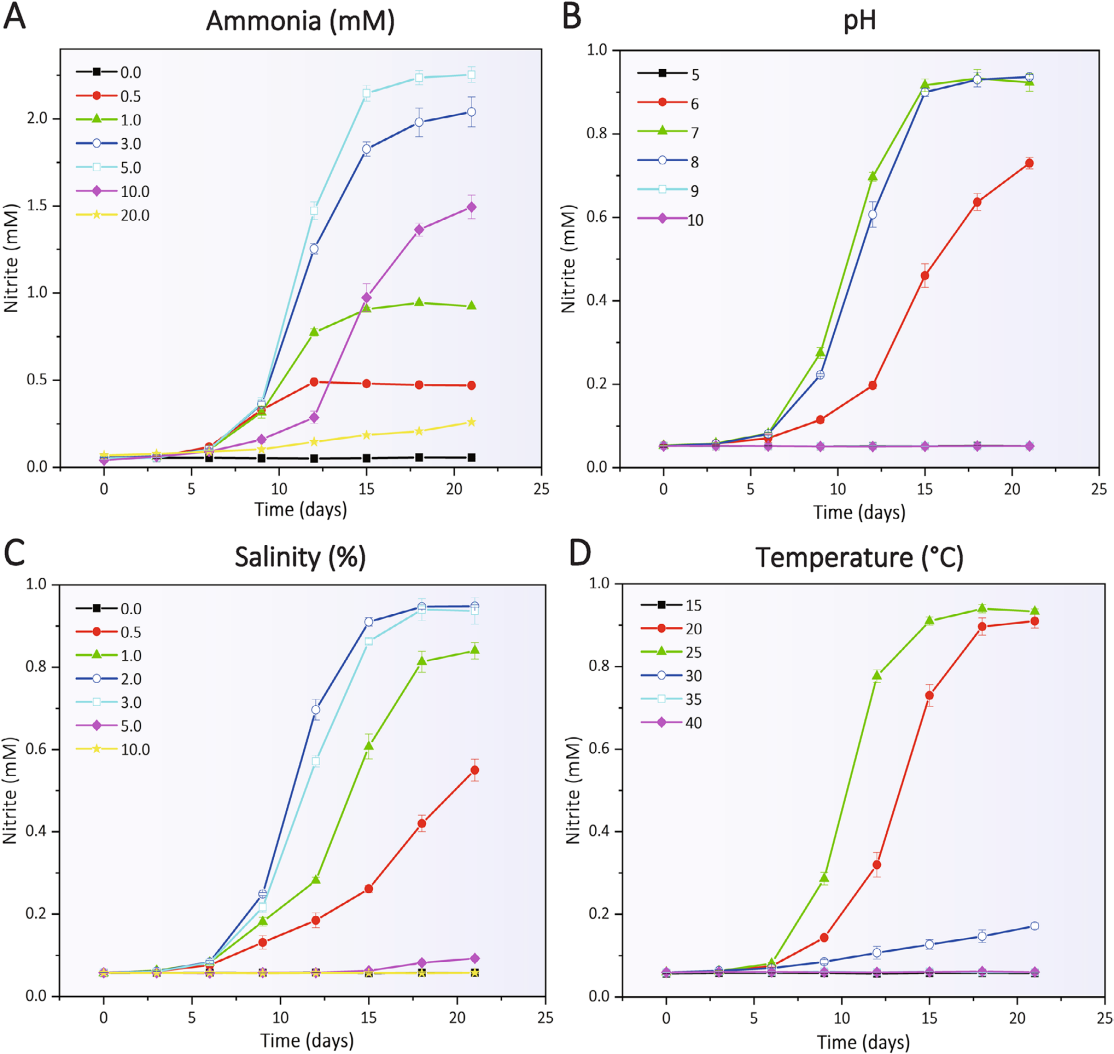
The effects of (A) ammonia, (B) pH, (C) salinity, and (D) temperature on the ammonia oxidation activity of strain CL1^T^. Each data point represents the average of three replicate experiments, with error bars indicating the standard deviations.

Strain CL1^T^ demonstrated a notable tolerance to ammonia, withstanding concentrations up to 10 mM, whilst its ammonia oxidation capability was markedly suppressed at 20 mM (Figure 4A). This resilience aligns with the characteristics of other documented AOA, including the MY1^T^, *Nitrosopumilus cobalaminigenes* HCA1^T^, and the SCM1^T^ (Jung et al. 2018; Qin et al. 2017) (Table 3). The strain exhibited optimal ammonia oxidation within a pH range of 7–8, with activity completely inhibited at pH levels below 6 or above 8 (Figure 4B). Similarly, the optimal salinity for ammonia oxidation was observed at 2%–3%, with no activity detected outside the range of 0.5%–3% (Figure 4C). Temperature‐wise, strain CL1^T^ achieved peak ammonia oxidation between 20°C and 25°C, with complete inhibition occurring below 20°C or above 30°C (Figure 4D).

These findings collectively indicate that strain CL1^T^ thrives under mesophilic, neutrophilic, and moderately halophilic conditions, with optimal growth parameters including an ammonia concentration of 0.5–1 mM, pH of 7–8, salinity of 2%–3%, and temperature range of 20°C–25°C. Notably, strain CL1^T^'s salinity tolerance distinguishes it from MY1^T^, which is adapted to low‐salinity environments with an optimal salinity range of 0.2%–0.4% (Jung et al. 2018) (Table 3). In contrast, strain CL1^T^, originating from a brackish habitat with a salinity of 2%–3%, exhibits a clear adaptation to marine conditions. This divergence in salinity preferences underscores the distinct ecological niches occupied by these strains, with strain CL1^T^ being particularly suited to the unique conditions of aquatic ecosystems.

Furthermore, strain CL1^T^'s salinity tolerance is consistent with other marine‐derived AOA, such as the HCA1^T^, *Nitrosopumilus oxyclinae* HCE1^T^ and *Nitrosopumilus ureiphilus* PS0^T^, which also display broad salinity adaptability. These strains maintain ammonia oxidation activity within salinity ranges of 1.5%–4.0%, 1.0%–4.0% and 1.5%–4.0%, respectively (Qin et al. 2017) (Table 3). Such findings highlight the general adaptability of marine AOA to varying salinity conditions, enabling them to sustain metabolic activity across diverse environmental settings. This adaptability underscores their ecological versatility and their critical role in nitrogen cycling within aquatic ecosystems.

Under optimal cultivation conditions—1 mM NH_4_Cl, pH 7, salinity 2%, and temperature 25°C—the 16S rRNA gene abundance of strain CL1^T^ increased by two orders of magnitude, rising from 2.54 × 10^7^ ± 8.89 × 10^5^ copies/mL to 3.55 × 10^9^ ± 3.36 × 10^8^ copies/mL over a 13‐day period (Figure 1A). Given that strain CL1^T^ encodes only a single copy of the 16S rRNA gene (as confirmed by genomic data), the copy number of this gene can be directly equated to cell count (Kim et al. 2012). This implies that the cell population of strain CL1^T^ theoretically increased by two orders of magnitude during this timeframe.

When compared to related ammonia‐oxidising archaea, such as the MY1^T^ and the SCM1^T^, strain CL1^T^ demonstrated superior ammonia utilisation efficiency. Specifically, the cell yields of the MY1^T^, the SCM1^T^, and strain CL1^T^ were estimated at 2.4 × 10^5^, 2.7 × 10^4^, and 3.92 × 10^6^ cells mL^−1^ μM ammonia^−1^, respectively. Additionally, strain CL1^T^ exhibited faster entry into the logarithmic growth phase, requiring only 7 days compared to 8 days for the MY1^T^ and 12 days for the SCM1^T^. Furthermore, strain CL1^T^ achieved a maximum specific growth rate of 0.87 d^−1^, significantly higher than the rates of 0.43 d^−1^ for the MY1^T^ and 0.78 d^−1^ for the SCM1^T^ (Könneke et al. 2005; Jung et al. 2018) (Table 3).

These findings highlight strain CL1^T^'s exceptional growth kinetics and ammonia utilisation efficiency, which surpass those of its close relatives, including *Nitrosarchaeum koreense* MY1^T^ within the same genus. The rapid entry into the logarithmic phase and the higher maximum specific growth rate make strain CL1^T^ an ideal model organism for future studies aimed at elucidating its physiology, metabolism, ecological role and evolutionary adaptations. These characteristics not only underscore strain CL1^T^'s potential as a key candidate for in‐depth research but also emphasise its significance in advancing our understanding of ammonia‐oxidising archaea in aquatic ecosystems.

### 3.4 Genome, Taxonomy and Potential Biological Features of Strain CL1^T^

A total of 414,630,524 raw reads were obtained through sequencing. After stringent quality control and assembly, a high‐quality genome sequence of approximately 1.63 Mb was generated. The completeness of the genome was assessed using CheckM v1.2.3 (Parks et al. 2015), revealing a completeness score of 99.95%, indicating a nearly complete genome. The mean guanine‐cytosine (G + C) content of the genome was calculated to be 32.84%. A low G + C content is often associated with adaptations to low temperatures, low oxygen levels, or other extreme environmental conditions (Mann and Chen 2010). Given that AOA are widely distributed in marine environments, their low G + C content may represent a key genomic strategy for thriving in oligotrophic marine habitats (Mann and Chen 2010). Using Prodigal v2.6.3, a total of 1946 open reading frames (ORFs) were predicted in the strain CL1^T^ genome, a number comparable to that of other marine AOA, such as *Nitrosopumilus maritimus* (Walker et al. 2010). The strain CL1^T^ genome also includes one 16S rRNA gene, one 5S rRNA gene, one 23S rRNA gene and 48 tRNA genes.

**FIGURE 5:**
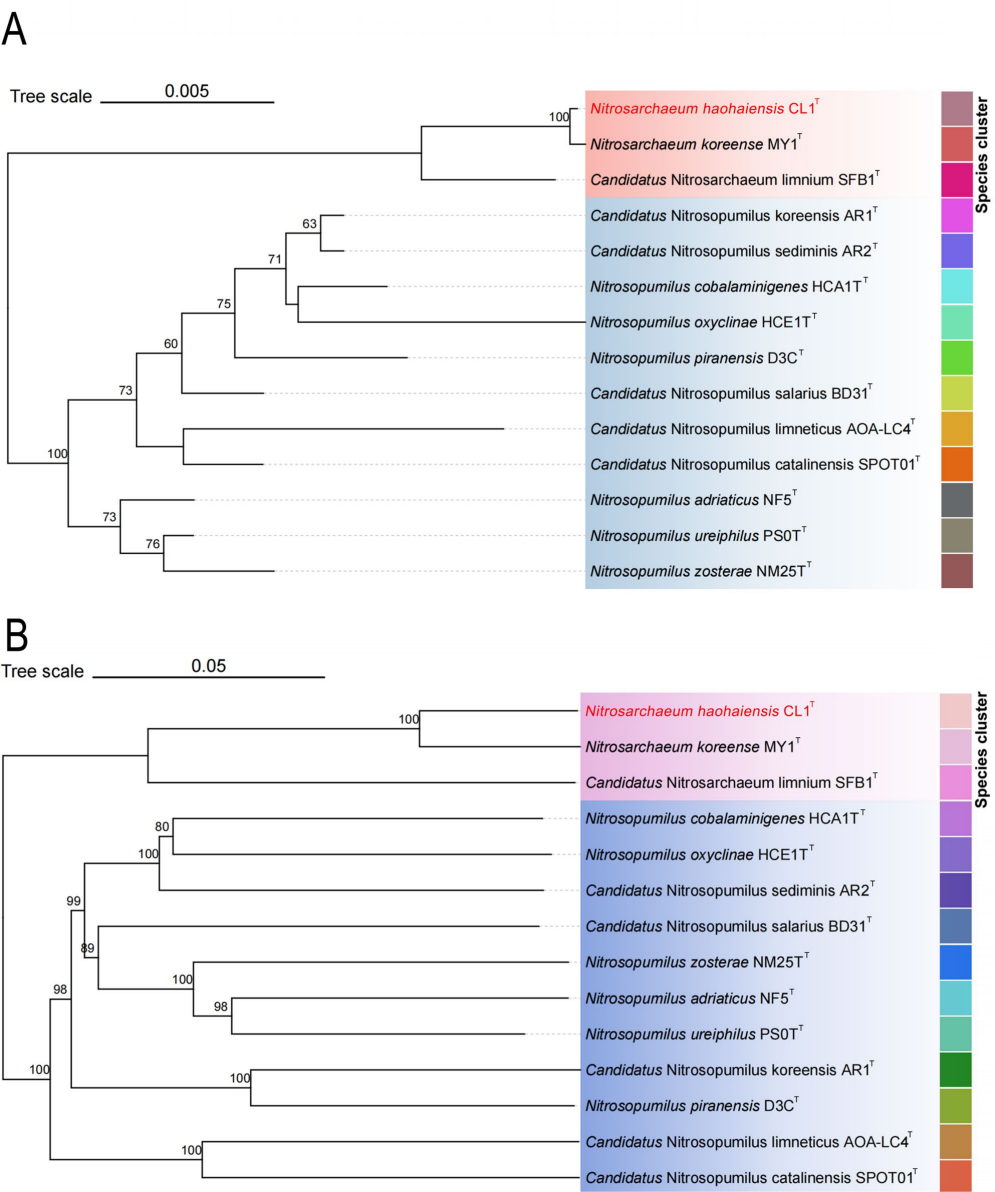
The phylogenetic trees illustrating the evolutionary relationships between strain CL1^T^ and reference species. The trees were constructed using FastME 2.1.6.1, based on the comparison of 16S rRNA gene sequences (A) and genome sequences (B), and inferred using the Generalised Time‐Reversible model with a gamma distribution (GTR + G). The branch lengths are scaled using the GBDP distance formula d5, with pseudo‐bootstrap support values exceeding 60% from 100 replications (average support: 71.5%). The trees were rooted at the midpoint and visualised with Chiplot. Colour blocks delineate different species groups. Strain CL1^T^ is highlighted in red.

**FIGURE 6:**
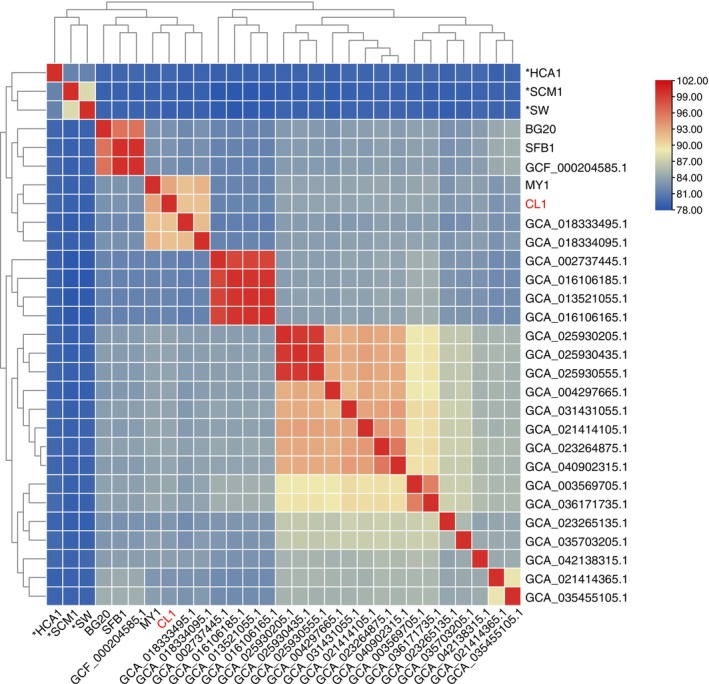
ANI matrix comparison of genomes amongst strain CL1^T^ and members of other *Nitrosarchaeum* and related *Nitrosopumilus*. Strain CL1^T^ is highlighted in red. HCA1^T^, SCM1^T^ and SW^T^ represent *Nitrosopumilus* strains available in pure culture and are marked with an asterisk, whilst the remaining entries correspond to high‐quality *Nitrosarchaeum* genome assemblies with > 85% completeness.

The taxonomic classification of strain CL1^T^ was confirmed through both phylogenetic tree construction and average nucleotide identity (ANI) analysis. The 16S rRNA‐based phylogenetic tree (Figure 5A) demonstrated that strain CL1^T^ forms a distinct branch with *Nitrosarchaeum koreense* MY1^T^, supported by a 100% bootstrap value, indicating a close evolutionary relationship. Similarly, the genome‐based phylogenetic tree (Figure 5B) clustered strain CL1^T^ within the *Nitrosarchaeum* genus, also with 100% bootstrap support, further validating its classification. Additionally, classification using GTDB‐TK (Genome Taxonomy Database Toolkit) placed strain CL1^T^ within the *Nitrosarchaeum* genus, with the highest ANI value of 92.01% when compared to the MY1^T^ (Figure 6). According to taxonomic standards, which define species within the same genus as having ANI values exceeding 95% (Tullio 2022), strain CL1^T^ represents a novel species of the *Nitrosarchaeum* genus, which we have designated *Nitrosarchaeum haohaiensis* (“Haohaiensis” is derived from the Latinized form of “Haohai,” which translates to “ocean” in Chinese, reflecting the East China Sea origin of the isolate. Additionally, “hao” and “hai” are components of the names of the researchers who isolated the strain, adding a personal and meaningful connection to the nomenclature. This dual significance highlights both the environmental source and the contributions of the isolators.). Strain CL1^T^ serves as the type strain of *Nitrosarchaeum haohaiensis*.

Despite the availability of numerous *Nitrosarchaeum* genome sequences obtained through metagenomic approaches, the majority of these strains have not been successfully cultured in pure form. Strain CL1^T^ represents only the second *Nitrosarchaeum* strain to be successfully cultured and is the sole pure culture derived from an aquatic environment. Genomic analysis further revealed that strain CL1^T^ possesses genes associated with adaptation to marine conditions, including the *ProP* gene, which aids in maintaining osmotic balance (Culham et al. 1993), and the *cutA* gene, which enhances tolerance to heavy metal ions (Fong et al. 1995). These genetic features underscore strain CL1^T^'s ability to thrive in brackish environments with salinities of 2%–3%.

A KEGG analysis revealed that strain CL1^T^ harbours the *amoA*, *amoB* and *amoC* genes, which encode the subunits of ammonia monooxygenase (AMO), the key enzyme responsible for catalysing the first step of ammonia oxidation (Stein 2019). Additionally, the KEGG annotation results demonstrated that strain CL1^T^ possesses multiple genes associated with ammonia assimilation pathways, including two major pathways: glutamate dehydrogenase (GDH) and glutamine synthetase‐glutamate synthase (GS‐GOGAT). Specifically, the genes *glnA_2* (NIOPMNAD_01859) and *glnA_1* (NIOPMNAD_00901) encode glutamine synthetase (EC 6.3.1.2, COG0174), a critical enzyme in the GS‐GOGAT pathway. Although this pathway is energy‐intensive, it is highly efficient for ammonia assimilation under low‐ammonia conditions (Tempest et al. 1970). In contrast, the gene *gluD* (NIOPMNAD_00511) encodes NAD‐specific glutamate dehydrogenase (EC 1.4.1.2), which is involved in the GDH pathway. This pathway is more efficient for ammonia assimilation under high‐ammonia conditions (Kanamori et al. 1987).

Through comprehensive comparative genomic analyses, we have uncovered a distinct phylogenetic distribution pattern of ammonia assimilation pathways amongst AOA. The GDH pathway is ubiquitously present across diverse AOA lineages, indicating its fundamental role in ammonia assimilation. In contrast, the GS‐GOGAT pathway displays a more specialised and restricted distribution, being confined to specific genera within the *Nitrososphaeraceae* family (e.g., *Nitrosocosmicus*) and select members of the *Nitrosopumilaceae* family (e.g., *Nitrosarchaeum* and *Nitrosopumilus*) (Wright and Lehtovirta‐Morley 2023). This limited phylogenetic distribution suggests that the GS‐GOGAT pathway may represent a more specialised and potentially evolutionarily conserved mechanism for ammonia assimilation in certain AOA lineages. The observed pattern raises intriguing questions about the evolutionary divergence and functional specialisation of ammonia assimilation strategies amongst AOA, warranting further investigation into the ecological and physiological implications of these distinct metabolic pathways.

Electron microscopy observations suggest that strain CL1^T^ cells likely secrete structures resembling extracellular vesicles (EVs) (Figure 2D,E). EVs are known to transport proteins, RNA, DNA, lipids and other small molecules, facilitating intercellular communication, material exchange and the expulsion of metabolic byproducts to maintain intracellular homeostasis (Maas et al. 2017). Although typical EV‐related protein‐coding genes, such as *arvA*, *arvB* and *arvC* (Mills et al. 2024), were not identified in the strain CL1^T^ genome, the presence of the ESCRT (Endosomal Sorting Complex Required for Transport) system core gene *vps4* was confirmed. The ESCRT‐III system is highly conserved across the *Thermoproteota* phylum and other members of the TACK superphylum, where it plays a critical role in membrane division and vesicle formation (Kuiper et al. 2024). This suggests that strain CL1^T^ may secrete EVs through an ESCRT‐III‐like mechanism. Additionally, the FtsZ protein, traditionally associated with cell division (Barrows and Goley 2021), was identified in strain CL1^T^. In the *Nitrososphaerota* phylum, FtsZ may also contribute to membrane remodelling or vesicle formation (Pelve et al. 2011). These findings open new avenues for investigating the mechanisms of EVs secretion in strain CL1^T^ and highlight potential molecular strategies employed by this archaeon to adapt to marine environments.

It is well‐established that pili and archaellum function as receptors for the attachment and infection of numerous archaeal viruses (Meier‐Kolthoff et al. 2013). However, electron microscopy observations of strain CL1^T^ revealed no extracellular structures resembling pili or archaellum (Figure 2D,E). Furthermore, genomic analysis failed to identify key genes associated with these appendages, such as *flaA* and *flaB* (archaellum genes) (Kühn et al. 2018) or *pilA* and *pilB* (pili‐related genes) (McCallum et al. 2017). Given the absence of these structures and their encoding genes, potential viruses infecting strain CL1^T^ are unlikely to rely on pili or archaellum for host recognition. Instead, other surface structures, such as the S‐layer, may play a critical role in viral attachment and infection.

**FIGURE 7:**
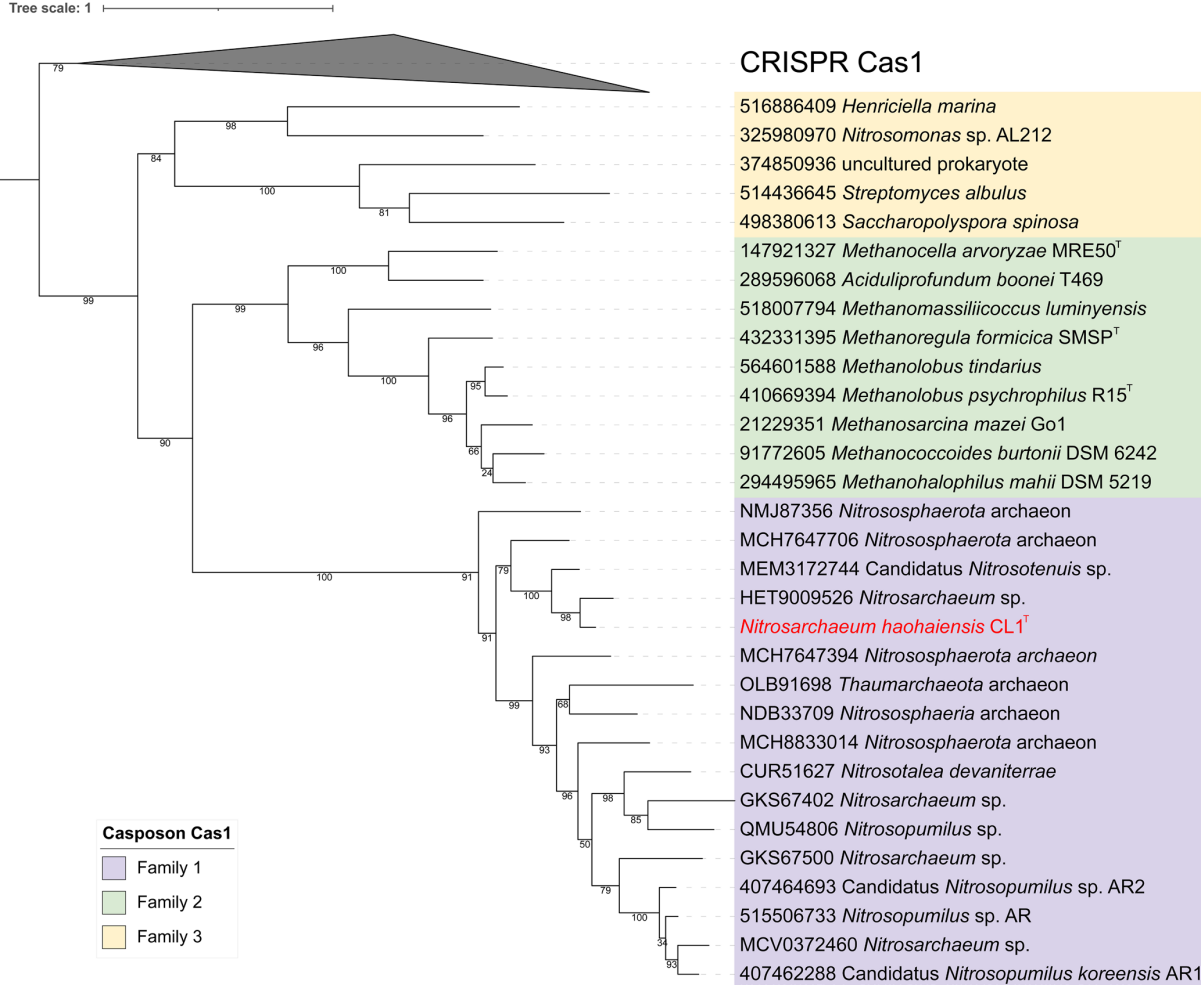
Phylogenetic tree of the Cas1 protein of strain CL1^T^. Related reference sequences were obtained from the NCBI database, and multiple sequence alignment was performed using MAFFT. The phylogenetic tree was constructed using FastTree and visualised using the Interactive Tree of Life (iTOL) website. The BLASTp hit sequences of strain CL1^T^'s Cas1 protein are represented by their NCBI accession numbers and strain names. Strain CL1^T^ is highlighted in red.

CRISPR‐Cas systems are a cornerstone of the immune defence mechanisms in prokaryotes, providing adaptive immunity against viral infections (Marraffini 2015). Predictive analysis using CRISPRCasFinder revealed that strain CL1^T^ lacks a complete CRISPR‐Cas system, which may increase its susceptibility to viral infections. This characteristic makes strain CL1^T^ an ideal model strain for studying virus isolation and host–virus interactions. Interestingly, despite the absence of a full CRISPR‐Cas system, the Cas1 protein (E‐value = 10–5, Query cover = 98%, Identity = 81.02%) was identified in the strain CL1^T^ genome. Cas1 is a key component of the CRISPR‐Cas system, typically involved in the acquisition and integration of new spacers into CRISPR arrays (Rath et al. 2015). Additionally, Cas1 has been shown to function as an integrase in casposons, a class of transposable elements associated with CRISPR systems (Krupovic et al. 2014). Phylogenetic analysis demonstrated that the Cas1 protein from strain CL1^T^ clusters within family 1 casposons (Figure 7), a unique group of proteins that appear to be exclusively associated with AOA. Notably, this clustering is particularly prominent in *Nitrosarchaeum* and *Nitrosopumilus* species, suggesting a specialised evolutionary relationship between these casposons and specific AOA lineages. This finding suggests that strain CL1^T^'s Cas1 may possess functional and evolutionary characteristics analogous to those observed in family 1 casposons (Krupovic et al. 2014), which utilise Cas1 for the integration of new DNA sequences into the genome.

Strain CL1^T^ has been identified to possess both the RM_type_I and RM_type_II restriction‐modification (RM) defence systems, which are widely distributed amongst bacteria and archaea (Gulati et al. 2024). These systems function as a protective mechanism by utilising restriction endonucleases to cleave foreign DNA and methyltransferases to methylate the organism's own DNA, thereby safeguarding it from invasive genetic material (Sitaraman 2016). However, unlike the CRISPR‐Cas system, RM systems provide only a basic level of defence, as their effectiveness is limited to the recognition of specific DNA sequences. This makes them less effective against highly variable viruses (Anton and Roberts 2023). Interestingly, we also identified a set of genes labelled as “pycsar_unknown,” which could not be classified under any known antiviral systems. These genes may represent a novel, previously uncharacterized defence mechanism, warranting further investigation to elucidate their functional roles and potential contributions to strain CL1^T^'s immune strategies.

Two transfer RNA (tRNA) genes, *tRNA‐Asp* (GTC) and *tRNA‐Ser* (TGA), were identified in the genome of strain CL1^T^. Beyond their primary role in protein synthesis, tRNA genes are frequently targeted as integration sites for mobile genetic elements, such as Integrative and Conjugative Elements (ICEs) and viral DNA (Beamud et al. 2024). This suggests that *tRNA‐Asp* (GTC) and *tRNA‐Ser* (TGA) in the strain CL1^T^ genome may function as potential hotspots for the integration of foreign genetic material. Further investigation is needed to explore the functional implications of these integration sites and their contribution to the genetic diversity and ecological adaptability of strain CL1^T^.

## 4 Conclusions

After a 3.5‐year enrichment cultivation process, a pure culture of a novel *Nitrosarchaeum* species was successfully isolated from the East China Sea, and its genome was sequenced. Comparative analysis revealed that strain CL1^T^, unlike the closely related *Nitrosarchaeum koreense* MY1^T^ and *Nitrosopumilus maritimus* SCM1^T^, exhibits a faster transition into the logarithmic growth phase and a higher maximum specific growth rate. The discovery of strain CL1^T^ not only expands our understanding of the diversity and physiological adaptability of ammonia‐oxidising archaea but also provides a valuable model for further research. Specifically, strain CL1^T^ offers a unique opportunity to investigate extracellular vesicles (EVs), viral interactions, and host–virus dynamics, as well as their roles and mechanisms in nitrogen cycling and ecosystem functioning.

## Author Contributions

H.L. isolated and cultured strain CL1^T^, conducted PCR, qPCR, FISH and EM, and wrote the manuscript. L.Z. performed genomic and phylogenetic analysis, and wrote the manuscript. H.C. isolated strain CL1^T^. Y.N. performed genomic sequence assembly. T.C. helped with configurations of software and scripts. L.C. and Y.Y. contributed to the conception. Y.W. designed and supervised the study, interpreted the data, and revised the manuscript. All authors read and approved the final manuscript.

## Conflicts of Interest

The authors declare no conflicts of interest.

## Data Availability

All study data are included in the article, and the 16S rRNA and *amoA* gene sequences of strain CL1^T^ have been deposited in GenBank under the accession numbers PQ498924 and SRR31438651, respectively.
